# Fistule réno-colique compliquant une tuberculose rénale: à propos d’un cas

**DOI:** 10.11604/pamj.2021.40.91.30518

**Published:** 2021-10-12

**Authors:** Ramzi Mejri, Kays Chaker, Mokhtar Bibi, Sami Ben Rhouma, Yassine Nouira

**Affiliations:** 1 Service d'Urologie, CHU Mongi Slim la Marsa, Tunis, Tunisie,; 2 Service d´Urologie, Hôpital La Rabta, Tunis, Tunisie

**Keywords:** Fistule réno-colique, tuberculose, néphrectomie, à propos d’un cas, Colorenal fistulas, tuberculosis, nephrectomy, case report

## Abstract

La tuberculose urogénitale reste peu évoquée et peu connue par les cliniciens. Les fistules réno-coliques sont parmi les formes compliquées de la tuberculose rénale survenant à un stade avancé de la maladie, mais qui sont rares. Elles intéressent en général le colon ascendant et descendant. Nous rapportons le cas clinique d´une patiente âgée de 58 ans qui s'est présentée aux urgences pour une pyélonéphrite aiguë gauche grave. La tomodensitométrie abdominale a objectivé une pyonéphrose gauche avec une fistule réno-colique gauche. Vu l´évolution clinico-biologique défavorable de la patiente malgré les mesures de réanimation et les traitements antibiotiques, une néphrectomie gauche a été pratiquée en urgence avec une déconnexion et un drainage dirigé de la fistule réno-colique. L'histologie a conclu à une tuberculose rénale. La patiente a été mise sous traitement anti-bacillaire selon le protocole 2ERHZ/4RH. Le traitement de cette forme de tuberculose doit être adéquat pour éviter toute possibilité de récidive dont la prise en charge serait alors plus compliquée.

## Abstract (en)

Urogenital tuberculosis is little suspected and known by clinicians. Colorenal fistulas, although rare, are complex forms of renal tuberculosis occurring in patients with advanced-stage disease. They generally occur in the ascending and descending colon. We here report the clinical case of a 58-year-old female patient presenting to the Emergency Department with severe acute left pyelonephritis. Abdominal CT scan objectified left pyonephrosis with left colorenal fistula. The patient had poor clinical, biological outcome despite resuscitation measures and antibiotic treatments. Urgent left nephrectomy was performed with disconnection and drainage of colorenal fistula. Histology showed renal tuberculosis. The patient received antibacillary drugs according to 2ERHZ/4RH guidelines. The treatment of this type of TB should be adequate to avoid recurrences that could be more complicated to manage.

## Introduction

La tuberculose est un problème de santé publique [1]. L'incidence de la tuberculose a considérablement augmenté ces dernières années surtout dans les pays en voie de développement. La localisation pulmonaire reste la plus fréquente, suivie de l'atteinte ganglionnaire et rénale [1,2]. En effet, L'atteinte rénale, généralement bilatérale, se fait par voie hématogène avec formation de granulomes glomérulaires. Ces lésions guérissent dans la plupart des cas sans produire de maladie rénale mais peuvent se compliquer par diverses pathologies infectieuses notamment la tuberculose rénale. Elle représente environ moins de 10% des fistules uro-digestives et nécessite dans la majorité des cas une néphrectomie [3].

## Patient et observation

**Information sur le patient**: une patiente de 53 ans, sans antécédents pathologiques notables, notamment pas de contage tuberculeux, consultait les urgences d´urologies pour des douleurs lombaires gauches fébriles évoluant dans un contexte d´anorexie et d´amaigrissement non chiffré.

**Résultats cliniques**: la patiente était fébrile à 39°C, tachycarde à 135 battements par minute. L´examen physique objectivait une nette défense de la fosse lombaire gauche. La pression artérielle était à 100/80 mm Hg.

**Figure 1:**
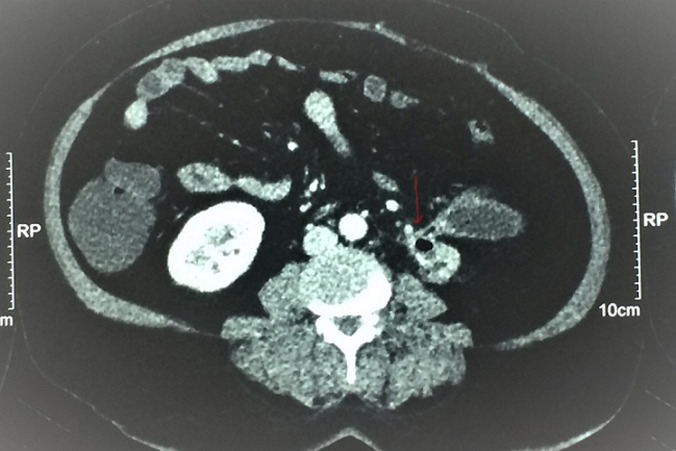
scanner abdominal avec injection de produit de contraste (coupe axiale) montrant un rein gauche de petite taille, à cortex très aminci, siège non seulement d´un calcul pyélique gauche de 15 mm mais aussi de plusieurs bulles d´air avec une collection péri rénale mesurant environ 70 mm

**Figure 2:**
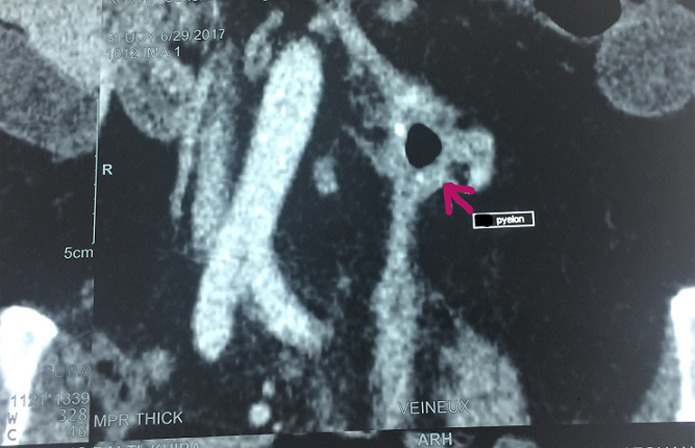
scanner abdominal avec injection de produit de contraste (coupe coronale) ; le colon gauche venant au contact avec le rein gauche avec passage de produit de contraste au niveau colique traduisant une fistule réno-colique

**Figure 3:**
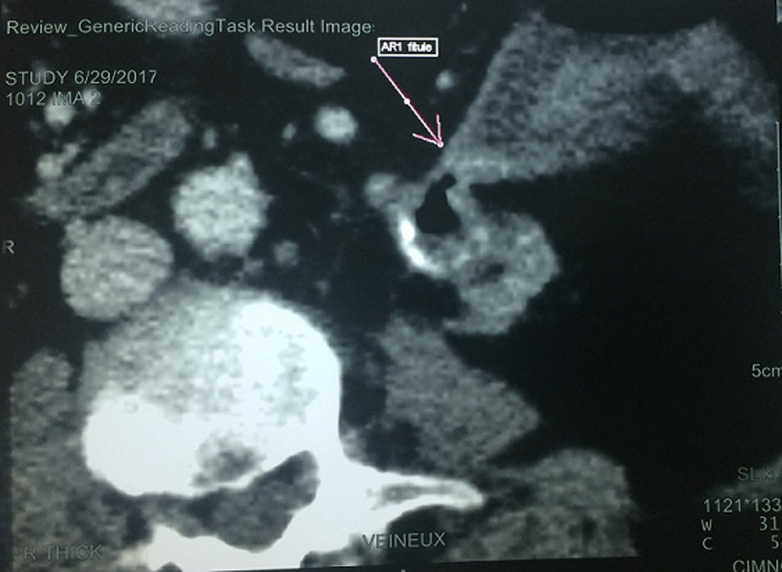
scanner abdominal avec injection de produit de contraste (coupe axiale); le colon gauche venait au contact avec le rein gauche avec passage de produit de contraste au niveau colique traduisant une fistule réno-colique

**Évaluation diagnostique**: sur le plan biologique, elle avait un syndrome inflammatoire avec une hyperleucocytose à 25000 Eléments/mm^3^et une CRP à 280 mg/l. La fonction rénale était normale. Les urines étaient purulentes et l´examen cytobactériologique des urines était positif à *E. Coli* sauvage. Une tomodensitométrie (TDM) abdominale montrait un rein gauche atrophique, à cortex très aminci, siège de multiples bulles d´airs au niveau des calices et un calcul pyélique de 15 mm (Figure 1). Une collection péri-rénale de 70 mm communiquait avec la voie excrétrice en dedans et le côlon gauche en dehors (Figure 1). Cette imagerie en coupe montrait aussi, un passage du produit de contraste au niveau du colon gauche témoignant la présence d´une fistule réno-colique (Figure 1, Figure 2, Figure 3). Le rein droit, les poumons, le foie, la rate ainsi que la vessie étaient normaux sur cet examen radiologique.

**Intervention thérapeutique**: la patiente avait reçu une antibiothérapie intra veineuse, faite d'une triple association (Céfotaxime, Gentamycine, Métronidazole). Devant la persistance du sepsis sévère après 72 heures de traitement bien conduit, une néphrectomie gauche associée à une ligature de l´uretère gauche était réalisée par une lombotomie antérolatérale gauche. Lors de l'exploration per opératoire le rein gauche était nettement diminué de taille avec une infiltration de l'espace péri rénal, comblé par du pus franc dont analyse bactériologique ne montrait pas de germes spécifiques. Une déconnexion de la fistule a été pratiquée associée à un large drainage selon la technique de Trémolières permettant une irrigation-drainage de la fistule prolongé jusqu´à 20 jours.

**Figure 4:**
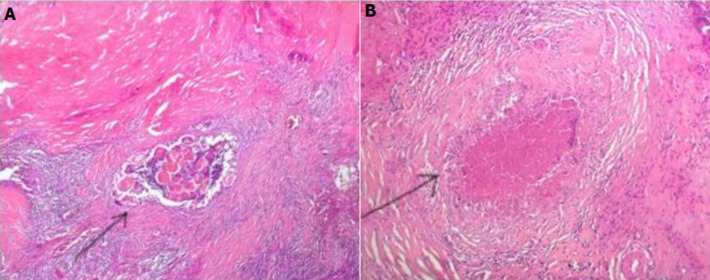
aspect histologique de la pièce opératoire de la néphrectomie gauche avec la coloration de l´hématoxyline éosine (HE * 200): une néphrite interstitielle chronique (A) avec granulomes épithélioïdes, gigantocellulaires et une nécrose caséeuse centrale caractéristique de la tuberculose rénale (B)

**Suivi et résultats**: les suites opératoires étaient marquées par le rétablissement de transit après 5 jours post opératoires et le tarissement complet de la fistule après 21 jours. L'examen histologique de la pièce opératoire concluait à une tuberculose rénale après avoir identifié des plages de nécrose caséeuse entourées de nodules épithélioïdes et gigantocellulaires (Figure 4). Ainsi, la patiente était mise sous traitement antituberculeux pris régulièrement pendant 6 mois. L´évolution était favorable sous traitement anti bacillaire selon le protocole 2ERHZ/4RH. Après un recul de 36 mois, la patiente est en bon état général avec une fonction rénale et un transit intestinal normaux.

## Discussion

En fait, les auteurs présentent un cas de fistule réno-colique, diagnostiquée à la tomodensitométrie abdominale chez une patiente de 53 ans avec un tableau de pyélonéphrite aiguë grave. La tuberculose est découverte à l'examen histologique de la pièce opératoire de la néphrectomie. La tuberculose est une maladie fréquente, en augmentation croissante, en particulier du fait de l'infection à VIH. La tuberculose urogénitale peut faire partie d'une infection disséminée ou être localisée uniquement au tractus urogénital. Les formes urinaires isolées sont d´évolution souvent lente et insidieuse. Les symptômes, aspécifiques, signent alors un stade avancé de l´infection. Les patients présentent le plus souvent un tableau de cystite chronique ou à répétition [1], se plaignant d´urgenturies et/ou de pollakiurie (4 à 88 % des patients selon les séries). Les douleurs lombaires (33 à 46 %), qui orientent vers une dilatation pyélocalicielle compliquant une sténose urétérale, et l´hématurie macroscopique (10 à 57 %) sont plus rares [2]. La tuberculose peut donner lieu à l´apparition de complications rares telles que les fistules réno-coliques [3]. Les fistules réno-digestives sont représentées essentiellement par les fistules réno-coliques (60% des cas) [4] et les fistules réno-duodénales (25% des cas) [4]. La fistule réno-colique est une complication très rare de diverses pathologies infectieuses, tumorales ou traumatiques du rein ou du colon [5]. La fistule réno-colique peut être une cause rare d'infection réfractaire des kystes rénaux [6]. L´origine de ces trajets fistuleux réno-digestifs est surtout rénale par des mécanismes obstructifs ou infectieux (tuberculose…), parfois une cause digestive (tumeurs malignes, maladie de Crohn, tuberculose…) ou plus rarement un traumatisme peuvent être retrouvés [5]. Selon la règle de Goodwin, les fistules hautes sont d´origine urinaire et les basses d´origine digestive [7].

Les fistules hautes sont exceptionnelles, le plus souvent d´origine inflammatoire chronique, secondaire à des calculs rénaux ou d´origine traumatique. Le diagnostic de la fistule colo-rénale doit être précoce, Il est basé sur des données cliniques et radiologiques. Les manifestations cliniques générales, digestives ou urinaires sont souvent polymorphes et non évocatrices [8]. Un cas de fistule réno-colique a été rapporté chez une femme obèse, diabétique qui s'est présentée dans un tableau de sepsis sévère, une détresse respiratoire d´installation brutale, une insuffisance rénale aigue et un diabète sucré déséquilibré [9]. La présence de fécalurie et ou de pneumaturie est pathognomonique mais reste rare [4]. La conservation du rein est pratiquement toujours impossible, la néphrectomie est le geste chirurgical le plus souvent réalisé. La fermeture de la fistule colique est réalisée par suture simple ou par résection anastomose du côlon qui peut être réalisée en un ou en deux temps [3,6,9]. La fistule digestive peut être prise en charge par d´autres techniques conservatrices. La technique mise au point par Trémolière [10] est une méthode quelle que peu contraignante. En effet, elle nécessite deux tubes: un tube d´aspiration (drain de gros calibre) positionné dans l´orifice fistuleux et un drain d´irrigation (drain de petit calibre) au voisinage du premier et un liquide d´irrigation fait d´ une solution d´acide lactique. Trois litres de cette solution doivent passer quotidiennement par un système d´irrigation. Un traitement adjuvant est à ajuster en fonction de l´étiologie [4]. L´adjonction d´un traitement antituberculeux est bien entendu impérative en cas de maladie tuberculeuse évolutive [3,9]. Pour les fistules survenant au cours d'une intervention percutanée sur le rein, le drainage par l´orifice de néphrostomie et de la voie urinaire supérieure par une sonde double J est généralement suffisant en l´absence de contamination péritonéale [11].

**Point de vue de la patiente**: durant son hospitalisation et après la fin du traitement, la patiente était ravie des soins qu'elle a reçus et parait optimiste quant à l'évolution de son état.

**Consentement éclairé**: il a été obtenu de la patiente pour que nous puissions utiliser les images. Il a volontairement donné son consentement éclairé pour permettre aux auteurs d'utiliser ses photos pour ce rapport de cas.

## Conclusion

Les fistules réno-coliques sont rares. Leur origine est le plus souvent rénale. Le traitement est pratiquement toujours une néphrectomie avec suture ou résection anastomose du segment digestif incriminé. Le bilan étiologique doit toujours comprendre la recherche d´une tuberculose même en cas de pathologie associée (calcul…). La fistule peut être en effet la seule manifestation clinique de cette maladie tuberculose. Ce diagnostic est souvent difficile à établir, mais la généralisation des techniques sérologiques et de biologie moléculaire devrait le faciliter.
